# Quantum Information and Foundations

**DOI:** 10.3390/e22010022

**Published:** 2019-12-23

**Authors:** Giacomo Mauro D’Ariano, Paolo Perinotti

**Affiliations:** QUIT Group, Dipartimento di Fisica dell’Università di Pavia, Istituto Nazionale di Fisica Nucleare, via Bassi 6, 27100 Pavia, Italy

**Keywords:** quantum information, quantum foundations, quantum theory and gravity

The new era of quantum foundations, fed by the quantum information theory experience and opened in the early 2000s by a series of memorable papers [1,2,3], led in a few years to a wealth of results, that can all be roughly traced back to the idea of testing quantum theory against new rivals instead of struggling in the worn-out attempt at its recomprehension within a classical imaginative world. The first remarkable construction of a toy theory for foundational purposes, in our knowledge, is represented by Ref. [4].

The study of foil theories along with their informational power lead to important progress, paralleled with an increasing understanding of the new foundational scenario [5]. Most importantly, this stream of thought is the origin of the new paradigm of the so-called *reconstructions*, which aim at singling out quantum theory in a wider scenario of possible theories of elementary physical systems [6,7]. Grant the authors an unwarranted bit of pride in stating that a clear picture of such a playground is now available thanks to the formulation of the concept of *Operational Probabilistic Theory* (OPT) [8,9]. As a result of the growing interest, we now understand quantum theory as a special kind of information theory, with postulates that regard the possibility or impossibility to carry out specific information processing tasks, instead of directly describing the mathematical structures of Hilbert spaces, operator algebras, and alike.

One of the future challenges for the informational approach to quantum foundations is then to embrace the mechanical part of the theory, besides the merely information-theoretic one, or, better, to remain on top of it.

The time demarcation represented by the year 2000 is of course artificial, just like every symbolic date, as quantum information was strictly connected to foundations since its very birth. One could not express this fact in better words than Chris Fuchs’ own: “*The title of the NATO Advanced Research Workshop that gave birth to this volume was ‘Decoherence and its Implications in Quantum Computation and Information Transfer’ …The life of the party was all the talks and conversations on ‘Decoherence and its Implications in Quantum Foundations’.*” [10]. The new approach, moreover, has some deep connections with the previous experience that can be broadly collected under the name *quantum logic*. Having said that, the turn of the century undoubtedly brought the foundations new vigour.

This special issue is meant to witness recent progress of the balanced and fertile interchange between the developments in application-oriented quantum information theory and those in foundations. As a result, the response of the authors was great, and produced a perfect blend of flavours. The subjects of the contributions can be briefly classified in three groups.

The first one can be deemed *resources*. One of the main topics in a well-organised information theory is quantification and classification of resources. It is nowadays common wisdom that *the* resource for quantum computation and information is entanglement, which is incidentally one of the main resources also for foundations. In a broader view, entanglement is one of the *nonclassical* resources allowed by quantum theory.

In the contribution, *Nonclassicality by Local Gaussian Unitary Operations for Gaussian States* [11], the authors introduce a measure of nonclassicality for Gaussian states of continuous variable systems and compare it with other measures of nonclassical correlations. The resource in this case is nonclassicality, namely, the ability to produce phenomena that are not reproducible by classical means. The proposed measure of nonclassicality is explicitly computed for a system of two bosonic modes, and estimated in the general case.

In another respect, one of the primary resources in quantum information is the ability to prepare states on demand. Methods for predicting the statistical efficiency of sources, or for sharpening our description of preparations through density matrices in the presence of partial information are then of the utmost importance.

In the paper, *Entropic Updating of Probabilities and Density Matrices* [12], the author analyses the task of reconstructing the theoretical description of a quantum state from partial experimental information. The standard relative entropy and the Umegaki entropy are derived in parallel from the same set of design criteria.

Finally, in the contribution, *Structure of Multipartite Entanglement in Random Cluster-Like Photonic Systems* [13], the authors analyse the size of multipartite entanglement in randomly generated cluster states, relating it to the density of nodes in the cluster.

A second collection of contributions regards *algorithms and protocols*. This selection witnesses progress in the ongoing challenge towards new algorithms and new tasks. In the contribution, *Finding a Hadamard Matrix by Simulated Quantum Annealing* [14], the author analyses quantum algorithms for finding a Hadamard matrix, which is itself a hard problem. The problem is reformulated in terms of energy minimisation of spin vectors connected by a complete graph, and approached via path-integral Monte-Carlo techniques. The scaling properties of the method show that the quantum algorithm outperforms its classical counterpart in solving this hard problem, providing yet another hint to quantum supremacy.

In the contribution, *Quantum Genetic Learning Control of Quantum Ensembles with Hamiltonian Uncertainties* [15], the authors propose a new method for controlling a quantum ensemble of two-level systems with uncertainties in the parameters of the Hamiltonian system. The method is based on the combination of a sample-learning control and a quantum genetic algorithm, witnessing the continuous cross-fertilisation between quantum theory and computer science.

The authors of the contribution, *Discrete Wigner Function Derivation of the Aaronson-Gottesman Tableau Algorithm* [16], present a discrete Wigner-function-based simulation algorithm for odd-d qudits that has the same time and space complexity as the Aaronson–Gottesman algorithm for qubits. The authors also discuss the differences between the Wigner function algorithm for odd-d and the Aaronson–Gottesman algorithm for qubits, conjecturing that they are due to the fact that qubits exhibit state-independent contextuality. This may provide a guide for extending the discrete Wigner function approach to qubits. Considering this result, one can easily realise how tightly quantum computation and quantum foundations are bound.

*Concepts and Criteria for Blind Quantum Source Separation and Blind Quantum Process Tomography* [17] discusses communication protocols for demixing a signal from the output of a communication line and establishes properties that were already used without justification in that context. The scenario considered here involves a pair of electron spins initially prepared in a pure state and then submitted to an undesired exchange coupling. The authors introduce a criterion for checking that the coupling does not produce entanglement.

In recent years, after studies that provided a fully algebraic method for analysing quantum circuits [18], it was realised that there are easy protocols challenging the circuit model, but are still amenable to a fully algebraic account [19]. Some of these protocols can be interpreted as computations that call events in a causally indefinite order, thus hinting to interesting foundational questions. In the article, *Non-Causal Computation* [20], the authors review recent results on indefinite orders and their potentiality in computation, replacing the requirement of a global ordering between gates in the computation with that of mere logical consistency.

The third collection regards *foundations*. This is the subject that encompasses all the remaining contributions, that amount to fifteen, with a very diverse span of subjects, approaches, and techniques.

One of the lessons of the quantum information theoretical approach to foundations is that very often physical concepts are easily grasped referring to the operations and processes they can undergo. In this spirit, the author of the contribution, *Agents, Subsystems, and the Conservation of Information* [21] proposes a mathematical modelling for subsystems of physical systems in the general scenario of OPTs, where subsystems are identified through a subalgebra of the full algebra of operations on the composite system they are part of. Various cases are then discussed, with a particular focus on quantum systems.

The relevance of appropriately treating subsystems of composite systems might appear somewhat technical at a superficial sight, but after giving the subject some more thought, one realizes that the notion of subsystem underlies many fundamental questions, e.g., Wigner’s thought experiment popularly known as the Wigner’s friend paradox. This is the subject of the contribution, *A No-Go Theorem for Observer-Independent Facts* [22], which proposes a perspective on the argument of Frauchiger and Renner [23] proving that “single-world interpretations of quantum theory cannot be self-consistent”. The author derives a no-go theorem for observer-independent facts, which would be common both for Wigner and the friend. This result is claimed to undermine one of the assumptions behind the concept of “self-consistency” by the authors of Ref. [23].

The analysis of conceptual foundational questions is possible thanks to the availability of a suitable mathematical language. A continuous process of reformulation and reconsideration of the latter is an important chapter in quantum foundations, as witnessed by the contribution, *A Royal Road to Quantum Theory (or Thereabouts)* [24]. Here, the author proposes an alternate perspective for approaching the problem of reformulating the mathematical language of quantum theory from simple postulates, based on the theory of Euclidean Jordan algebras. While the paper, as declared by the author, “fails to derive quantum mechanics”, it derives a more general framework that embraces the quantum along with alternate, not wildly different possible theories.

In addition, the article *Quantum Theory from Rules on Information Acquisition* [25] reviews a reconstruction of the mathematical framework of quantum theory. The starting point here is a set of rules constraining an observer’s acquisition of information about physical systems. The reconstruction offers an informational explanation for entanglement, monogamy, and nonlocality, from limited accessible information and complementarity. The analysis leads to a notion of “conserved informational charges” that stems from complementarity relations that characterise the unitary group and the set of pure states.

The review *The Many Classical Faces of Quantum Structures* [26] addresses a mathematical reformulation of quantum mechanics in terms of classical mechanics. The standpoint for this approach is that interpretational problems with quantum mechanics can be phrased precisely by only talking about empirically accessible information. This review spells out the main points of the abovementioned approach in terms of the algebraic structures lying behind quantum theory.

After the reconstruction of the mathematical language of quantum theory from information theoretical postulates was completed, one of the possible developments was the attempt at a reformulation of quantum mechanics from information processing. In this respect, much progress was achieved, essentially showing that one can have a fully information-theoretic account of the basic equations at the core of relativistic quantum field theory, such as Weyl’s and Dirac’s [27,28], and Maxwell’s [29]. The next difficult step in this direction is introducing interactions. A recent result in this direction is the study of all possible interacting cellular automata in one dimension along with a full diagonalization of their two-particle sector [30]. In the contribution, *Solutions of a two-particle interacting quantum walk* [31], the authors provide an alternative solution of the dynamics of the abovementioned class of cellular automata based on a path-sum approach.

Once again, on the exploration of the language of quantum foundations, one can read *Ruling out Higher-Order Interference from Purity Principles* [32], where the authors analyse the principles of Causality, Purity Preservation, Pure Sharpness, and Purification in the operational framework of generalised probabilistic theories, proving that these principles limit interference to second-order, namely, the interference pattern formed in a multislit experiment is a function of the interference patterns formed between pairs of slits. This behaviour is typical of quantum theory, where there are no genuinely new features resulting from considering three slits instead of two. Systems in such theories correspond to Euclidean Jordan algebras.

Another contribution that is focused on the mathematical language and its framework is *Leaks: Quantum, Classical, Intermediate and More* [33], where the authors introduce the notion of a leak for general process theories and identify quantum theory as a theory with minimal leakage, as opposed to classical theory that has maximal leakage. Leaks are processes that provide leakage of classical information, and can be introduced in most theories. These processes allow for a category theoretical account of decoherence as a mechanism for the emergence of classical theory in a quantum scenario. The authors also discuss the relation of leaks with purity of processes.

One of the main themes in the context of reconstructions and reformulations of quantum theory is to open the route to possible new post-quantum theories. The article, *Iterant Algebra* [34] moves a step beyond quantum theory, starting from a generalisation of the structure of matrix algebra, motivated by the structure of measurement for discrete processes. Iterant algebra is shown to embrace matrix and Clifford algebras, and the framework is then applied to discuss various aspects of quantum mechanics, such as the Schrödinger and Dirac equations, Majorana Fermions, and representations of the braid group.

We now move to a different chapter in foundations, where one can use the standard mathematical formalism to face questions and concepts that have interpretational issues. An example is given by *Robust Macroscopic Quantum Measurements in the Presence of Limited Control and Knowledge* [35]. The authors tackle the problem of compatibility of quantum behaviour and macroscopic measurements, focusing on the estimation of the polarization direction for a large system of spin 1/2 particles. The analysis starts from a model of von Neumann pointer measurement and shows traits of a classical measurement for an intermediate coupling strength. A relevant part of the contribution is devoted to the analysis of response of the model against relaxations of the initial assumptions, showing that the model is robust.

One of the fundamental subjects that attracted interest from the very birth of quantum mechanics is uncertainty. The study of uncertainty is still lively, and the present special issue includes one contribution that is devoted to this subject: *Measurement Uncertainty Relations for Position and Momentum: Relative Entropy Formulation* [36]. The authors analyse uncertainty as related to incompatibility of different observables, where the latter is quantified by the amount of unavoidable approximation in a joint measurement. As a quantifier of information loss, the authors consider relative entropy of a “true” probability distribution and an approximating one. Such an analysis is applied to obtain lower bound for the amount of information that is lost by replacing the distributions of the sharp position and momentum observables, as they could be obtained with two separate experiments, by the marginals of any smeared joint measurement.

The renewed interest in fundamental problems produced new approaches to the unification of quantum mechanics and the theory of gravity. Recent trends in quantum gravity are thus of high interest for the community working in foundations and, for this reason, we appreciate the value of a contribution such as *Planck-Scale Soccer-Ball Problem: A Case of Mistaken Identity* [37], which reports about reflections on the rule of composition for momenta. Over the last decade, nonlinear laws of composition of momenta were predicted by many approaches to quantum gravity. In order to dissipate concerns about such nonlinearity, the author discusses the subtle difference between the two roles that a law of momentum composition play: the first one is related to the description of space-time locality, and the second one is related to translational invariance. The contribution exhibits an example of space-time where the local structure provides a nonlinear composition of momenta and yet translational invariance is expressed by a linear law for the addition of momenta of many-particle systems.

Another contribution focused on a model aiming at a formulation of quantum gravity is *Entropic Phase Maps in Discrete Quantum Gravity* [38], where the author makes an attempt based on path summation over a space of evolutionary pathways in a history configuration space. This approach enables derivation of discrete Schrödinger-type equations, and mathematical constructions thereof are used to introduce entropic functions that obey an abstract version of the second law of thermodynamics.

One of the most remarkable consequences of the widespread interest in foundations is a flourishing of experiments aimed at testing fundamental questions, or challenging established pillars of quantum theory. A remarkable example is the Pauli exclusion principle for Fermions, that has been tested in a series of recent experiments, in an ongoing effort that is witnessed also by a contribution in the present issue, *Test of the Pauli Exclusion Principle in the VIP-2 Underground Experiment* [39]. Here, the authors report progress of the VIP-2 experiments at the Laboratori Nazionali del Gran Sasso, seeking a prohibited transition in copper atoms of a 2*p* orbit electron to the fully populated ground state, via X-ray analysis. The present limit on the probability for Pauli exclusion principle violation for electrons set by the VIP experiment is $4.7\times 10^{-29}$. A first result from the VIP-2 experiment improves on the VIP limit, while the goal is a gain of two orders of magnitude in the long run.

A second example is the test of spontaneous collapse models, which aim at an objective solution of the measurement problem that keeps the quantum formalism untouched while tweaking its dynamical equations. In the contribution, *CSL Collapse Model Mapped with the Spontaneous Radiation* [40], new upper limits on the parameters of the Continuous Spontaneous Localization collapse models are extracted. The main idea behind the experiment is to analyse IGEX data about X-ray emission and compare them with the spectrum of the spontaneous photon emission process predicted by collapse models. This study allows for the exclusion of a broad range of the parameter space for CSL models.

Finally, we include a contribution out of line, which is more focused on interpretational issues than technical, such as *Quantum Information: What Is It All About?* [41]. In this contribution, the author answers the provocative question originally posed by John Bell, claiming that, in the consistent histories approach to quantum theory, information is meant about projectors on subspaces of the Hilbert space of a system, representing its quantum properties. The main focus is the discussion of how the single-framework rule—i.e., the rule for assigning probabilities to a projective decomposition of the identity—for consistent histories avoids contradictions and recovers both classical information theory and macroscopic physics. Room for issues is left only in the regimes without classical analogue, where a single framework is not sufficient.

As a concluding remark, we would like to thank all the authors for their contributions and declare our satisfaction in verifying the ongoing interest in fundamental problems—the only possible fuel for the science and technology of tomorrow.
